# Patriotism, nationalism, and evaluations of the government’s handling of the coronavirus crisis

**DOI:** 10.3389/fpsyg.2023.1016435

**Published:** 2023-02-06

**Authors:** Yida Zhai, Yizhen Lu, Qidi Wu

**Affiliations:** Shanghai Jiao Tong University, Shanghai, China

**Keywords:** governmental performance, lockdown, nationalism, patriotism, the COVID-19 pandemic, threat perceptions

## Abstract

**Introduction:**

The COVID-19 pandemic has been accompanied by a global rise of nationalism, and many countries’ responses to the pandemic have further intensified nationalist sentiments. The public is polarized over government policies toward the pandemic. Hence, this study examined the associations of patriotism and nationalism with the support for lockdown policies and evaluations of governmental performance.

**Methods:**

We recruited 180 participants from one Chinese university.

**Results:**

Patriotism and nationalism had a direct effect on evaluations of governmental performance. Patriotism also had indirect effects on favorable evaluations of governmental performance through the support of lockdown policies. In addition, this study examined the relationship of threat perception and evaluations of governmental performance in the pandemic.

**Discussion:**

This relationship was found to be insignificant; however, the interaction effects between threat perception and patriotism on evaluations of governmental performance were significant. The implications of the study results are discussed.

## Introduction

The spread of coronavirus disease 2019 (COVID-19) has brought the state into the spotlight again (Woods et al., 2020). Individual states continue to be the principal actors that take measures to tackle problems brought about by the pandemic, such as protection, treatment, vaccine development, setting restrictions, and the closure of borders. To mitigate the spread of the pandemic, states have sought to restrict people’s movement and international trade. Sometimes, efforts to fight the pandemic come at the expense of international cooperation. For example, countries may impose export restrictions on medical supplies to ensure local availability; protectionist policies further isolate one country from the other (Baldwin and Freeman, 2020; Evenett, 2020; Mistreanu, 2020). Many observers contend that the pandemic has fueled nationalism globally (King, 2020; Rachman, 2020; Tisdall, 2020; Vogel, 2020). In particular, governments’ nationalist policies in response to the pandemic aggregate nationalist sentiments (Bieber, 2020; Su and Shen, 2021).

The COVID-19 pandemic has triggered a series of crises that have altered the environment in which politics works. It is widely acknowledged that patriotism and nationalism are thriving amid the crisis that creates a threat to people living in the same community (Hutchinson, 2017; Su and Shen, 2021). These two orientations indicate people’s attachment to and identification with nation-states as their ingroup. The crisis creates fear and uncertainty among people. Individuals seek to reduce uncertainties and anxiety by increasing attachment to their ingroup; countries provide security and safety to people in return for their loyalty and commitment (Druckman, 1994). In intergroup relations, a search for scapegoats, exclusion, and biases are associated with a rise of patriotism and nationalism amid the crisis. Moreover, patriotism and nationalism do not always take a radical manner, but can also exist in daily lives in unremarkable forms, intervening in people’s ways of thinking unconsciously (Antonsich, 2020). Crises can amplify the influence of patriotism and nationalism.

This study examined the associations of patriotism and nationalism with attitudes toward the Chinese government’s responses to the pandemic. To contain the spread of the virus, the government had to implement lockdown policies. Undoubtedly, there were debates and opposition to such a policy. The Chinese government employed the rhetoric of a war against the coronavirus. Through appealing to patriotism and nationalism, the government wanted citizens to comply with its policies and obtain their collaboration. Did patriotism and nationalism generate the public’s support for lockdowns? Did lockdown policies help increase favorable evaluations of governmental performance in response to the pandemic? In addition, we are interested in the associations of patriotism and nationalism with evaluations of governmental performance in handling the coronavirus crisis. Clearly, favorable evaluations are critically relevant for incumbent administrations and political leaders during crises such as the COVID-19 pandemic.

Moreover, we examined the associations of threat perception with evaluations of governmental performance in handling the coronavirus pandemic. Perceived threat is a critical factor that influences people’s attitudes toward an ingroup (Lavine et al., 2005; Moskalenko et al., 2006; Sinkkonen and Elovainio, 2020; Zhai, 2022a). External circumstances such as crises may exacerbate the existing perceived threat from an outgroup. Under the influence of threat perception, patriotism and nationalism tend to rise (Klingeberg, 1983; Nincic and Ramos, 2012). To some extent, threat from outgroups provides a catalyst to increasing the cohesion of an ingroup. We predicted that perceived threat from the United States would increase people’s favorable evaluations of the Chinese government’s performance. Moreover, perception of threat influences the relationship between patriotism/nationalism and attitudes toward ingroups or outgroups (Ljujic et al., 2013; Caricati, 2018). We examined the interaction effects between patriotism and threat perception and between nationalism and threat perception on evaluations of governmental performance in handling the pandemic.

Patriotism involves individuals’ attachment to their country. It reflects positive feelings in the relationship between the persons and their country, which are the foundation for the existing state. Patriotism usually possesses commendatory connotations, indicating a noble and honorable sentiment (Nincic and Ramos, 2012). However, affection toward a country does not entail hating others. Social psychologists have found long ago that the categorization of an “us” and “them” would generate ingroup favoritism but only produce outgroup hostility under certain conditions (Allport, 1954; Brewer, 1999, 2000; Weisel and Böhm, 2015; Zhai, 2017). As early as the time of Floyd Allport and George Herbert Mead, social psychologists have sought to distinguish patriotism from nationalism; the latter was viewed as a major cause of war (Kosterman and Feshbach, 1989). Love of one’s own country is a form of healthy genuine patriotism. In contrast, *pseudopatriotism* values blind conformity to authority and the exclusion of other nations as outgroups (Adorno et al., 1950). Patriotism is a healthy national self-concept and is associated with a cooperative or peaceful approach to the world; patriotic orientations may occur in non-competitive situations (Druckman, 1994). Hence, patriotism is viewed as positive, benign feelings.

The perceived threat from outgroups can trigger a rise of patriotic feelings (Klingeberg, 1983; Nincic and Ramos, 2012). Under a rising wave of attachment to one’s country, people become willing to put the interests of their country over their own individual interests. Because of the benefits of mobilizing patriotism, politicians often employ it for the sake of the incumbent leadership (Coryn et al., 2004; Kam and Ramos, 2008; Lakoff, 2008; Gustafsson, 2016). Politicians even intentionally confuse patriotism and unwavering support for state power and authority. In history, patriotism has been associated with events where individuals engaged in bloody, violent behavior justified in the name of national interests. Politicians tend to overstate dangers around every corner to arouse fear among people, by which they would be able to ask the public for their commitment and loyalty to political authority in the name of their love of country.

Nationalism is a way of thinking that values membership in a nation rather than other groups and understands the nation-state as the natural unit of social and political organization; hence, the state is made to be congruent with the nation (Fox and Miller-Idriss, 2008; Bieber, 2020). Nationalism provides legitimacy to the nation-state system in world politics (Mayall, 1990; Woods et al., 2020). The personal identity of members of a nation constitutes an essential and relevant element of nationalism (Greenfeld, 1992). Divisions of nations can reinforce ingroup solidarity (Hutchinson, 2006). In daily life, people actively invoke and organize ideas of nation and national identity in social interactions (Thompson, 2001; Fox and Miller-Idriss, 2008). Civic and ethnic nationalism are its two basic forms (Smith, 1991; Greenfeld, 1992). In studies on ethnic politics, researchers have found that ethnic identity tends to glorify one’s own group and vilify others (Schertzer and Woods, 2021). Perception of threat from other countries is a factor that fosters this trend. Nationalism not only creates favorable sentiments for one’s nation, but also rouses hatred and negative resentment against other foreign nations (Kleinpenning and Hagendoorn, 1993; Woods et al., 2020).

Political leaders regularly appeal to patriotism and nationalism to mobilize their countries for national efforts in response to crises (Nincic and Ramos, 2012). During the COVID-19 pandemic, some populist politicians have inspired patriotism and nationalism to shape their countries’ response and policies for the pandemic. So far, much of world politics occurs in the nationalist context. Patriotism and nationalism are ingrained into most societies around the world (Bieber, 2020). They intertwine with government responses and policies and shape people’s attitudes toward economic, social, and political issues. Hence, it would be meaningful to examine their role in the global coronavirus pandemic.

The present study aimed to examine the associations of patriotism and nationalism with people’s evaluation of their governments’ performance in handling the coronavirus crisis. In the face of the pandemic, government policies suspended or reduced individual freedoms and civil liberties to some extent, which caused resistance and opposition to these policies among many. Emergencies typically provide politicians the opportunity to extend their power and intervene in citizens’ freedoms. Hence, some researchers are concerned about a rise in authoritarianism as a consequence of the pandemic (Bieber, 2020). To earn public support and cooperation, governments may mobilize patriotism and nationalism and use these to obtain the public support they may need.

*Hypothesis 1*: Patriotism and nationalism are positively associated with favorable evaluations of the government’s response to the COVID-19 pandemic.

The public’s evaluations of the government’s performance are related to its policies adopted in response to the pandemic. On January 23, 2020, the Chinese government placed the 11 million residents of Wuhan city under lockdowns, and 2 days later, the whole of Hubei province was locked down. Nationwide traffic restrictions reduced and stopped inter-city travel and intra-city activities and forced the self-isolation of the population at home (Tian et al., 2020; Yuan et al., 2020). With the global spread of the virus, many other countries have also adopted similar lockdown policies. Understandably, people would be unhappy if they were told to stay home or could not move freely. There is a heated debate over lockdown policies. Supporters state that such a policy helps control the spread of the virus and, therefore, save people’s lives. Opponents, on the other hand, advocate that such a policy violates their freedoms and liberties and has little effect in controlling the pandemic. Patriotism and nationalism are two orientations that link individuals to their country. Psychological dynamics in an ingroup shape people’s attitudes toward the group’s policy. Therefore, patriotism and nationalism may affect public attitudes toward lockdown policies. In addition, as a situational factor, people’s attitudes toward lockdown policies may also affect their evaluations of governmental performance. If lockdowns can contain the virus, its positive effect may cause the public to favorably evaluate governmental performance. During the pandemic, the rocketing numbers of confirmed cases and the death toll in other countries triggered Chinese nationalism to revolve around pride for their apparent success in controlling the crisis (Woods et al., 2020). We will examine the direct and indirect effect of patriotism and nationalism on evaluations of governmental performance through an investigation of people’s attitudes toward lockdown policies.

*Hypothesis 2*: Patriotism and nationalism are positively associated with support for lockdown policies.

*Hypothesis 3*: Patriotism and nationalism indirectly affect evaluations of the government’s response to the COVID-19 pandemic through individuals’ support for lockdown policies.

Moreover, a perception of threat from outgroups is a relevant factor in influencing attitudes toward ingroups. Previous studies have demonstrated that perception of outgroup threat contributes to increasing ingroup cohesion and solidarity (Huddy et al., 2005; Skitka, 2005; Greenaway and Cruwys, 2019). The coronavirus crisis triggered several real and perceived threats in inter-state relations; among these, the most remarkable was that derived from the conflict between China and the United States (Woods et al., 2020; Zhai, 2022a). Before and during the pandemic, the United States had been engaged in a trade war with China and had taken a series of sanctions on Chinese companies. As a result of the Chinese government’s propaganda, an increasing number of Chinese people believe that the United States seeks to impede China’s development and impose a great threat to its national security (Sinkkonen and Elovainio, 2020). U.S. government officials have also harshly criticized China’s policies on the pandemic, arguing that China was responsible for the worldwide spread of COVID-19. Despite WHO guidelines regarding the name of the virus, then-President Donald Trump regularly called the virus the “Wuhan virus” or “Chinese virus,” instead of the neutral “COVID-19”; United States officials repeatedly claim that the virus was leaked from a Chinese laboratory rather than through the natural environment (Pompeo, 2020; Rogers et al., 2020). China has regarded these unverified arguments as intentions seeking to tarnish China’s image and pushed back all criticisms (Zhao, 2020a,b). Previous studies demonstrate that the presence of external threats increases a person’s level of patriotism and nationalism (Klingeberg, 1983; Nincic and Ramos, 2012). The COVID-19 pandemic is no longer merely a public health crisis but a question of national security. The United States’ blame on China is perceived as a threat to the country’s national security. National-security hawks will consequently have more favorable evaluations of their government. Studies have indeed shown that unfavorable evaluations of the US’ response to the COVID-19 pandemic significantly predicted Chines people’s attitudes toward the superiority of their country’s political system (Zhai, 2022b). Moreover, perceptions of threat from the United States may interact with patriotism and nationalism and influence Chinese people’s evaluations of their government’s performance in handling the coronavirus crisis.

*Hypothesis 4*: Perceptions of threats from the US moderate the relationship of patriotism and nationalism with evaluations of the government’s response to the COVID-19 pandemic.

## Materials and methods

### Participants

We recruited participants from one Chinese university. Undergraduate students who took the course “Introduction to Political Science” were invited to participate in this study. Participants filled out an internet-based survey questionnaire in class during break time. Participants were offered monetary compensation after completing the survey. Finally, we recruited 180 participants (53% males, 47% females). The participants ranged from 17 to 25 (*M* = 19.54, SD = 1.15) years old.

### Measures

#### Patriotism

Kosterman and Feshbach’s (1989) patriotism scale was adapted and used to measure patriotism. The sample items were as follows: “I love my country;” “I am proud to be Chinese;” “It is not that important for me to serve my country;” and “When I see the Chinese flag flying, I feel great.” Responses were coded on a 5-point Likert scale (1 = strongly disagree to 5 = strongly agree). Greater scores indicate higher levels of patriotic feelings (*M* = 4.52, SD = 0.64). Cronbach’s alpha was 0.84.

#### Nationalism

Kosterman and Feshbach’s (1989) nationalism scale was adapted and used to measure nationalism. The sample items were as follows: “The important thing for the Chinese foreign aid program is to see to it that China gains a political advantage;” “Other countries should try to make their government as much like ours as possible;” and “The Chinese nation has the greatest history and culture in the world.” Responses were coded on a 5-point Likert scale (1 = strongly disagree to 5 = strongly agree). Greater scores indicate higher levels of nationalism (*M* = 3.86, SD = 0.71). Cronbach’s alpha was 0.63. Other studies have also reported Kosterman and Feshbach’s (1989) nationalism scale as having a relatively low validity (Dekker et al., 2003).

#### Support for lockdown policies

Participants were asked to state their attitudes with regards to supporting lockdown policies amid the COVID-19 pandemic. Responses were rated on a 5-point Likert scale (1 = strongly disagree to 5 = strongly agree). Higher scores indicate more favorable attitudes about the policy (*M* = 4.39, SD = 0.85).

#### Evaluations of the government’s performance

Participants reported how favorably they evaluate the government’s performance in handling the coronavirus crisis on a seven-point scale. Responses were rated on a 5-point Likert scale (1 = strongly unfavorable to 5 = strongly favorable). Higher scores indicate more favorable evaluations of the government’s performance in handling the crisis (*M* = 4.34, SD = 0.69).

#### Threat perception

Threat perception was measured using the item “The United States is a threat to China’s development.” Responses were coded on a 5-point Likert scale, ranging from 1 “strongly disagree” to 5 “strongly agree.” Greater scores indicate higher levels of threat perception (*M* = 3.79, SD = 1.11).

### Ethics consideration

The respondents’ participation in this study was voluntary and they were assured that all data would be anonymous and used only for research purposes, and that participation would not cause any physical or psychological harm to them. Their informed consent for participation was obtained prior to the start of the survey, and they were free to stop answering the study questionnaire at any time.

### Data analysis

The order of analysis was a correlation analysis, a test of the mediation effect, and a test of the moderation effect. We conducted multivariate regression analyses. The calculation was performed using STATA (version 12). For all tests, a significance level was set at 0.05.

## Results

Correlations among the variables are presented in Table 1. Both patriotism and nationalism were positively correlated with support for lockdown policies (*r* = 0.47, *p* < 0.001; *r* = 0.39, *p* < 0.001). In addition, patriotism and nationalism were positively correlated with favorable evaluations of governmental performance (*r* = 0.48, *p* < 0.001; *r* = 0.49, *p* < 0.001). Support for lockdown policies was positively correlated with favorable evaluations of governmental performance (*r* = 0.55, *p* < 0.001).

**Table 1:** Means, standard deviations, and zero-order correlations between the variables.

Variables	*M*	SD	1	2	3	4	5	6	7
1. Age (years)	19.54	1.15	1.00						
2. Gender (female = 1)	0.47	0.50	**0.16**	1.00					
3. Patriotism	4.52	0.64	0.05	**−0.18**	1.00				
4. Nationalism	3.86	0.71	0.09	**−0.16**	**0.61**	1.00			
5. Support for lockdown policies	4.39	0.85	0.06	−0.09	**0.47**	**0.39**	1.00		
6. Threat perceptions	3.79	1.11	0.01	0.01	0.06	0.02	**0.16**	1.00	
7. Evaluations of governmental performance	4.34	0.69	0.11	0.11	**0.48**	**0.49**	**0.55**	0.09	1.00

Bold font indicates the correlation coefficients are statistically significant at the 5% level (two-tailed test).

**Figure 1:**
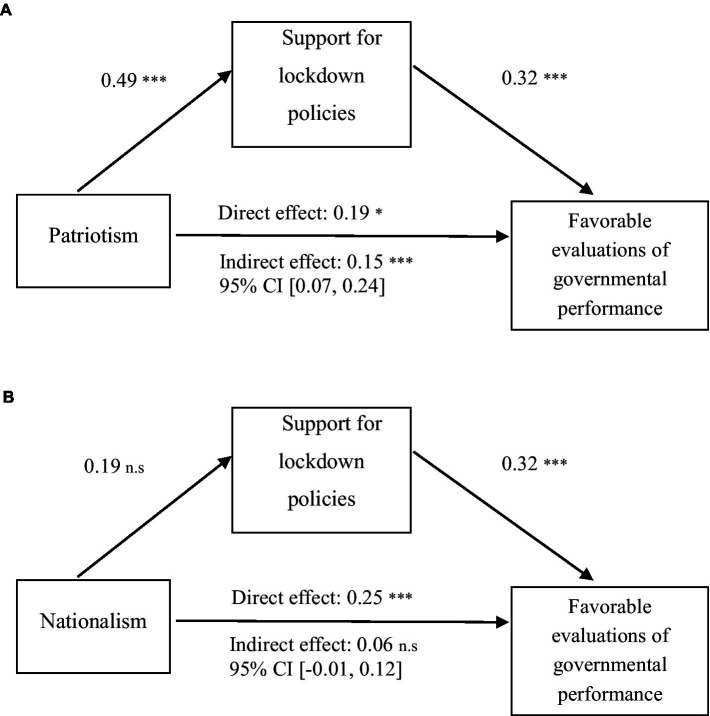
Path model of the effects of patriotism and nationalism on favorable evaluations of government performance, *p < 0.05, ***p < 0.001.

We examined the mediation effects of supporting attitudes toward lockdown policies on the associations of patriotism and nationalism with favorable evaluations of governmental performance. Figure 1A shows that patriotism directly predicted favorable evaluations of governmental performance (*β* = 0.19, SE = 0.08, *p* < 0.05). In addition, patriotism was positively associated with support for lockdown policies (*β* = 0.49, SE = 0.11, *p* < 0.001); people who were supportive of lockdown policies tended to favorably evaluate governmental performance. Hence, patriotism had an indirect effect on favorable evaluations of governmental performance through supporting attitudes toward lockdown policies (*β* = 0.15, SE = 0.04, *p* < 0.001).

Figure 1B shows that nationalism only had direct effects on favorable evaluations of governmental performance. Nationalism positively predicted favorable evaluations of governmental performance (*β* = 0.25, SE = 0.07, *p* < 0.001). However, nationalism was not significantly associated with support for lockdown policies (*β* = 0.19, SE = 0.10, *p* > 0.05). Hence, the indirect effect of nationalism on evaluations of government performance was insignificant (*β* = 0.06, SE = 0.03, *p* > 0.05).

Table 2 displays the relationships of threat perception and evaluations of governmental performance in handling the pandemic. Threat perception was not significantly associated with favorable evaluations of governmental performance (*β* = 0.05, SE = 0.04, *p* > 0.05). The interaction item of threat perception and patriotism was significantly associated with favorable evaluations of governmental performance (*β* = −0.12, SE = 0.05, *p* < 0.01, Δ*R*^2^ = 0.08), while the interaction item of threat perception and nationalism was non-significant (*β* = −0.03, SE = 0.05, *p* > 0.05).

**Table 2:** The effect of patriotism, nationalism, and threat perceptions on evaluations of the government’s performance during the COVID-19 pandemic.

Dependent variables	Evaluations of governmental performance	
	*β* (SE)	*β* (SE)
Age	0.02	0.01
	(0.04)	(0.04)
Gender (Female = 1)	0.29**	0.32***
	(0.09)	(0.08)
Patriotism	0.22***	0.17**
	(0.05)	(0.05)
Nationalism	0.22***	0.22***
	(0.05)	(0.05)
Threat perceptions	0.05	0.05
	(0.04)	(0.04)
Patriotism × Threat perceptions		−0.12**
		(0.05)
Nationalism × Threat perceptions		−0.03
		(0.05)
Intercept	3.88***	4.09***
	(0.73)	(0.69)
*R* ^2^	0.34	0.42
Δ*R*^2^		0.08
*N*	180	180

^**^p < 0.01, ^***^p < 0.001, two-tailed test.

**Figure 2:**
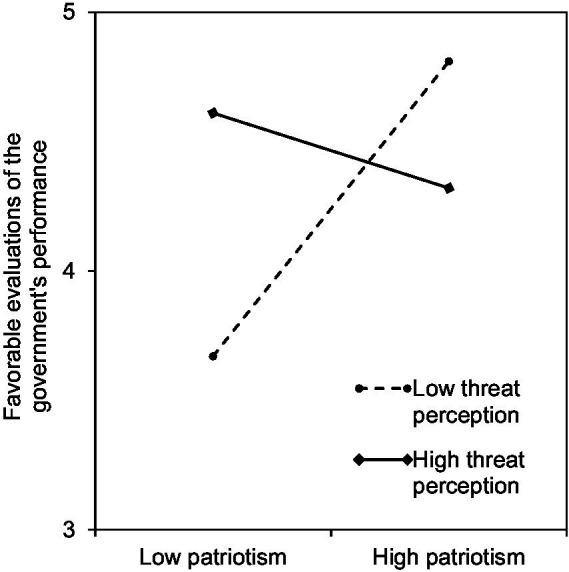
Interaction effects of patriotic feelings and treat perceptions on favorable evaluations of the governments performance, *p* < 0.01.

Figure 2 plots the interaction effect of threat perception and patriotic feelings on evaluations of governmental performance in handling the coronavirus crisis. When threat perception was low (−1 SD), people who had high levels of patriotism tended to favorably evaluate governmental performance. When the perception of threat was high (+1 SD), people with low patriotism tended to have a better evaluation of governmental performance, while favorable evaluations of governmental performance declined among people who had higher levels of patriotism.

## Discussion

Nation-states continue to play an indispensable role in the contemporary world, and the concepts such as national sovereignty and territories are still relevant to international relations (Zhai, 2021). Patriotism and nationalism revitalize and become critical factors in a country’s domestic politics and international relations. The Chinese government has led a campaign of patriotism and nationalism over the past few decades (Zhao, 1998; Brady, 2002). As a result, patriotic and nationalist Chinese people show a tendency of excluding foreign cultures and upholding the current political system (Tang and Darr, 2012; Cheng et al., 2017; Wang and Kobayashi, 2021).

This study examined the mechanism in which patriotism and nationalism affect public evaluations of the Chinese government’s response to the pandemic. Attitudes toward lockdown policies are a mediator between political ideology and evaluations of governmental performance. Even though lockdown policies and other measures to restrict people’s freedoms and civil liberties adopted amid the pandemic may be authoritarian according to some observers (Woods et al., 2020), the Chinese government advertises their effects in containing the disease. Support for lockdown policies is mainly derived from patriotism rather than nationalism. People who support such a policy tend to make favorable evaluations of governmental performance in handling the crisis. The results show that favorable evaluations of governmental performance are influenced by the feelings of one’s nation being superior to others and feelings of love for the country. Although patriotism and nationalism were both associated with favorable evaluations of governmental performance, nationalism had a greater effect than patriotism. Viewing the group in positive terms (“better” than another group) enhances members’ self-esteem; nationalism links individuals’ self-esteem to the esteem of the nation (Druckman, 1994). They are not only part of a good nation, but their nation is “better” than others. Nationalism entails national superiority, and such feelings lead to better evaluations of one’s own country’s responses to the pandemic.

Attitudes toward lockdown policies were positively associated with favorable evaluations of governmental performance. Through support for lockdown policies, patriotism and nationalism had an indirect effect on evaluations of governmental performance. In general, Chinese people have high levels of favorable evaluations of their government’s responses to the coronavirus crisis. The Chinese government managed to build mobile cabin hospitals in a shorter time, administer more nucleic acid tests, produce more medical supplies, and organize a large-scale quarantine. As a result, China rapidly contained the spread of the virus domestically and achieved economic revival in 2020 (He, 2020; Tanzi and Lu, 2020). In comparison with other countries that are still suffering from a worsening situation, patriotism and nationalism buttress Chinese citizens’ favorable evaluation of their government’s performance. In our interview, respondents expressed their confidence in the Chinese political system, which is viewed to have an “advantage” in responding to emergencies such as the pandemic crisis. The potential implication of the pandemic on Chinese society is that ordinary citizens rationalize and favor increased state power and intervention. This trend is associated with a rise in patriotism and nationalism. The pandemic reinforces the perception of the superiority of the Chinese system to the western democratic model, which has a long-term influence on China’s political development.

The effects of threat perception on evaluations of governmental performance in handling the COVID-19 pandemic were insignificant. Even though the perception of threats from an outgroup (i.e., the United States) was high among the respondents, the results show that their favorable evaluations of governmental performance were not derived from a perception of threat. Death anxiety has been found to be a driving force for people’s attitudes toward the nationalist policies of a government (Su and Shen, 2021). Death anxiety could be a primary reason for support for lockdown policies and favorable evaluations of governmental performance. Patriotism interacted with threat perception, and their interaction effects on evaluations of governmental performance were significant. Interestingly, perceived threat from outgroups only increased favorable evaluations of governmental performance among people who had low levels of patriotism. Conversely, with a rise in perception of threat, favorable evaluations of governmental performance declined among those who expressed high patriotic feelings.

The results indicate a double face for those who chant patriotic rhetoric. These “patriots” like to overtly express their love of country and favor of government policies. However, when they perceive a formidable threat from outgroups, they change their favorable evaluations of the government radically. As patriotism has a positive connotation, both political elites and ordinary people express love for their country. However, displays of patriotism could be performative, showing symbolic traits of patriotism but without substance. Ironically, patriotism could be linked with rioters. For example, Donald Trump enjoys talking about patriotism, and his daughter Ivanka Trump called her father’s supporters who violently stormed the United States Capitol in January 2021 as “American patriots.” Moreover, these pro-Trump people believed themselves to be true patriots, and their attack on the capitol, protection of the country (Adams, 2021). These facts indicate ideological characteristics of patriotism. People unify under the banner of patriotism, but the substance of love for the country can be understood in different ways. High-profile patriots could be fence-sitters and least patriotic. As shown in our study, people shift to make negative evaluations of the domestic government when perceiving formidable external threats.

Some limitations in this study should be acknowledged. First, the measure of nationalism was less satisfactory. Cronbach’s alpha was 0.63 for nationalism. As the scale of nationalism developed by Kosterman and Feshbach (1989) was based on the circumstances of the US, this study reveals a problem in its application in other cultures such as China. Second, this study primarily focused on China. Stringent lockdown policies and the official discourse on the threat of the US deeply make sense for the Chinese case. However, the findings regarding the relationship between patriotism, nationalism, the perception of threats from outgroups, and support for lockdown policies should be further tested in other countries.

## Conclusion

This study examined the effects of patriotism and nationalism on people’s attitudes toward government responses to the coronavirus crisis and the mechanism in which such effects take place. In the post-pandemic world, patriotism and nationalism will intertwine closely and shape people’s attitudes toward society and politics. These two orientations increase people’s loyalty to ingroups and help governments dismiss opposing opinions. Even though there are debates over the suppression of individual freedoms by lockdowns, governments can obtain public support through mobilizing citizens’ patriotic and nationalist sentiments. In addition, patriotism had indirect effects on favorable evaluations of governmental performance through attitudes of endorsement of lockdown policies. The results indicate that the mobilization of patriotism and nationalism is important for authorities to obtain favorable evaluations of governmental performance in handling the coronavirus crisis.

Moreover, support for lockdown policies was closely related to favorable evaluations of governmental performance. Even though patriotism and nationalism had significant effects on people’s evaluations of governmental performance, comparing these dispositional factors, situational factors had greater effects. When people perceived the necessity of adopting lockdown policies to prevent the spread of the virus, support for such a policy directly contributed to favorable evaluations of governmental performance.

Authorities take advantage of actual or fabricated outgroup threats to consolidate their legitimacy. In the case of the coronavirus crisis, we found that perception of threat from an outgroup did not significantly increase favorable evaluation of domestic authorities. As the Chinese government obtained high levels of favorable evaluations of its performance among citizens, the results indicate that the favorable evaluations were not from concerns about outgroup threat but had alternative reasons. For example, support for lockdown policies had great positive effects on evaluations of governmental performance.

Investigating the interaction effects between patriotism and threat perception and between nationalism and threat perception generated new findings. The results indicate that propaganda on outgroup threat and subsequent increase in threat perception generated double effects. For those who had low patriotic feelings, perceptions of outgroup threat can increase their favorable evaluations of governmental performance. However, a perception of increasing outgroup threat will undermine favorable evaluations of governmental performance among those who had high patriotic feelings. A perception of a formidable threat offsets an affection for a country sustained by patriotism.

## Data availability statement

The raw data supporting the conclusions of this article will be made available by the authors, without undue reservation.

## Ethics statement

Ethical review and approval was not required for the study on human participants in accordance with the local legislation and institutional requirements. The patients/participants informed consent for participation was obtained prior to the start of the survey, and they were free to stop answering the study questionnaire at any time.

## Author contributions

YZ: conceptualization, methodology, formal analysis, original draft preparation, and reviewing and editing. QW: conceptualization, methodology, investigation, and reviewing and editing. YL: conceptualization, methodology, investigation, and reviewing and editing. All authors contributed to the article and approved the submitted version.

## Conflict of interest

The authors declare that the research was conducted in the absence of any commercial or financial relationships that could be construed as a potential conflict of interest.

## Publisher’s note

All claims expressed in this article are solely those of the authors and do not necessarily represent those of their affiliated organizations, or those of the publisher, the editors and the reviewers. Any product that may be evaluated in this article, or claim that may be made by its manufacturer, is not guaranteed or endorsed by the publisher.
